# Functional constraints on adaptive evolution of protein ubiquitination sites

**DOI:** 10.1038/srep39949

**Published:** 2017-01-05

**Authors:** Liang Lu, Yang Li, Zhongyang Liu, Fengji Liang, Feifei Guo, Shuai Yang, Dan Wang, Yangzhige He, Jianghui Xiong, Dong Li, Fuchu He

**Affiliations:** 1 State Key Laboratory of Proteomics, Beijing Proteome Research Center, Beijing Institute of Radiation Medicine, 27 Taiping Road, Beijing 100850, China; 2 National Center for Protein Sciences Beijing, 38 Life Science Park Road, Beijing 102206, China; 3 State Key Laboratory of Space Medicine Fundamentals and Application, China Astronaut Research and Training Center, 26 Beiqing Road, Beijing 100094, China; 4 Space Institute of Southern China, 3 Pingdi Industrial Road, Shenzhen 518117, China

## Abstract

It is still unclear whether there exist functional constraints on the evolution of protein ubiquitination sites, because most previous studies regarded all protein ubiquitination sites as a whole or only focused on limited structural properties. We tried to clarify the relation between functional constraints and ubiquitination sites evolution. We investigated the evolutionary conservation of human ubiquitination sites in a broad evolutionary scale from *G. gorilla* to *S. pombe*, and we found that in organisms originated after the divergence of vertebrate, ubiquitination sites are more conserved than their flanking regions, while the opposite tendency is observed before this divergence time. By grouping the ubiquitination proteins into different functional categories, we confirm that many functional constraints like certain molecular functions, protein tissue expression specificity and protein connectivity in protein-protein interaction network enhance the evolutionary conservation of ubiquitination sites. Furthermore, by analyzing the gains of ubiquitination sites at different divergence time and their functional characters, we validate that the emergences of ubiquitination sites at different evolutionary time were also affected by the uncovered functional constraints. The above results suggest that functional constraints on the adaptive evolution of ubiquitination sites increase the opportunity for ubiquitination to synthetically regulate various cellular and developmental processes during evolution.

Proteins ubiquitination at lysine residues mediates 80~85% of the protein degradation in eukaryotic cells, and this ATP-dependent process is efficient and highly specific^1^,^2^. Ubiquitination is involved not only in protein degradation, but also in a broad spectrum of cellular processes including cell cycle progression^3^, apoptosis^4^, transcriptional regulation^5^, DNA damage repair^6^ and immune response^7^. Disorder of ubiquitination is found to be related with various human diseases, such as cancer^8^,^9^,^10^ and neuronal disorders^11^,^12^,^13^. The specificity of ubiquitination sites extends the functions of ubiquitination, making it possible to regulate various biological processes. Exploring the evolution of ubiquitination sites and functional constraints would be helpful to understand the underlying mechanism and function of ubiquitination.

Taking 753 ubiquitination sites of 626 proteins from public database as a whole, Hagai *et al*.^14^ analyzed ubiquitination sites evolution and found that ubiquitination sites are weakly more conserved than unmodified lysine residues, and ubiquitination sites tend to evolve faster in disordered regions than in ordered domains. By analyzing 281 ubiquitination sites of 252 proteins that first appeared along the human lineage, Kim *et al*.^15^ found that these novel ubiquitination sites could be involved in the evolution of protein degradation and other regulatory networks. However, as ubiquitination can be involved in a wide range of biological functions, the functional constraints might also influence the evolutionary conservation of the ubiquitination sites. As previous studies regarded all ubiquitination sites as a whole in a limited evolutionary period or only focused on their structural propensity, it remains to be explored whether there exists a relation between functional constraints and ubiquitination sites adaptive evolution.

To acquire more accurate and comprehensive understanding of the relation between functional constraints and ubiquitination sites evolution events, we first investigated the evolutionary conservation of the human ubiquitination sites in a broad evolutionary scale by aligning them with their orthologs from *G. gorilla* to *S. pombe* (Fig. 1) and we found that in organisms originated after the divergence of vertebrate, ubiquitination sites are more conserved than their flanking regions, while the opposite tendency is observed before this divergence time; then, by grouping the ubiquitination proteins into different functional categories, we also found that many functional constraints like certain functions (such as enzyme binding, cellular macromolecule metabolic process and developmental process), protein tissue expression specificity and protein connectivity in protein-protein network enhance the evolutionary conservation of ubiquitination sites; finally, by analyzing the gains of ubiquitination sites at different divergence time and their functional characters, we validates that the emergence of ubiquitination sites at different evolutionary time were affected by the uncovered functional constraints. Above results suggest that functional constraints on the adaptive evolution of ubiquitination sites increase the opportunity for ubiquitination to synthetically regulate various cellular and developmental processes during evolution.

## Results and Discussion

### Conservation of ubiquitination sites versus their flanking regions over a broad evolutionary scale

**Figure 1:**
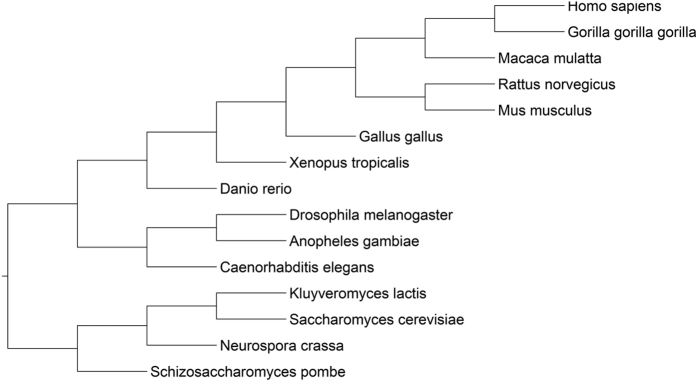
Phylogeny of the organisms for ubiquitination sites evolution analysis. The phylogeny of these organisms was obtained by the tool of Taxonomy Common Tree from NCBI^59^, and the phylogenetic tree was plotted using FigTree (http://tree.bio.ed.ac.uk/software/figtree/).

Previous studies pointed out that ubiquitination sites are more conserved compared with their flanking regions^14^, however, it is still unclear how the evolutionary rate of ubiquitination sites changes at different evolutionary periods. To answer this question, we investigated the evolutionary conservation of human ubiquitination sites (Supplementary Table S1) in a broad evolutionary scale by aligning them with their orthologs in 14 common model organisms: *G. gorilla, M. mulatta, R. norvegicus, M. musculus, G. gallus, X. tropicalis, D. rerio, D. melanogaster, A. gambiae, C. elegans, K. lactis, S. cerevisiae, N. crassa* and *S. pombe* (Fig. 1). Then we introduced Poisson distance^16^ to estimate the evolutionary conservation of ubiquitination sites. The Poisson distance can correct multiple substitutions at a site and has the linear relationship with time^16^, and this strategy has been successfully used to measure the evolutionary conservation of phosphorylation sites^17^. Following Wang *et al*.^17^, we selected the ten flanking residues around a ubiquitination site other than other lysine residues as the background to correct the potential biases^17^,^18^ (see Materials and Methods).

Then we performed z-score test to examine whether there exists significant difference between the Poisson distance of ubiquitination sites and that of flanking regions in each individual reference organisms. In addition to obtaining the *P*-value for each individual reference organisms through z-score test, we employed chi-square test to obtain an overall *P*-value to assess the significance of the difference between the Poisson distance of ubiquitination sites and that of flanking regions across multiple reference organisms (see Materials and Methods).

**Figure 2:**
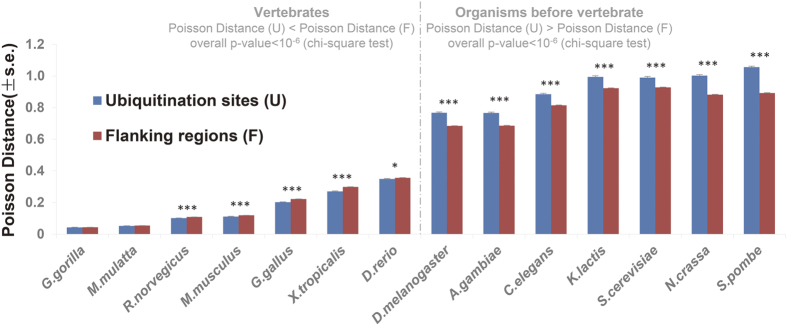
Comparison of the Poisson distance between ubiquitination sites and their flanking regions (s.e., standard error; *P-value < 0.05; **P-value < 0.01; ***P-value < 0.001). 14 reference organisms were arranged along the X axis in the order of their evolutionary distance to *H. sapiens*. The dashed line indicates the divergence time of vertebrate. In organisms originated after the divergence of vertebrate (on the left side of the dashed line), ubiquitination sites are more conserved than their flanking regions, while the opposite tendency is observed before this divergence time (on the right side of the dashed line). U: ubiquitination sites; F: flanking regions.

To obtain novel finding for the evolutionary tendency of ubiquitination sites, we adopted more ubiquitination datasets and reference organisms in a broad evolutionary scale. We observed that using individual reference organism before the divergence of vertebrate, ubiquitination sites evolved faster than flanking regions, while using individual reference organism after the divergence of vertebrate, ubiquitination sites are more conserved than flanking regions. Moreover, we calculated overall *P*-value for multiple reference organisms, and we also observed significant differences. On the overall level, ubiquitination sites evolve faster before the vertebrate divergence time, while after that, ubiquitination sites are more conserved (Fig. 2), which is consistent with analysis in individual reference organism. This finding suggests that there might be some functional constraints increasing the opportunity for fine regulation of the ubiquitination-mediated cellular and developmental processes during evolution^19^, which leads to the promotion in evolutionary conservation of ubiquitination sites.

### Constraints of various biological factors on ubiquitination sites evolution

Constraints shape phenotypic evolution together with adaption^20^. Although the basis of the mechanism for phenotypic evolution proposed by Darwin (1859) was adaptation^21^, it is recognized that the response to natural selection is subject to various constraints that place qualitative and quantitative limits on the course or outcome of adaptive evolution^22^,^23^,^24^,^25^,^26^,^27^. These constraints can be biomechanical-chemical, developmental, genetic and functional. As for proteins, systematic surveys indicate that protein evolution is not determined exclusively by selection on biological processes, but also affected by their expression patterns and their position in biological networks. In this study, we tried to explore what functional constraints influence ubiquitination sites conservation by classifying proteins into various categories.

#### KEGG pathway categories

**Figure 3:**
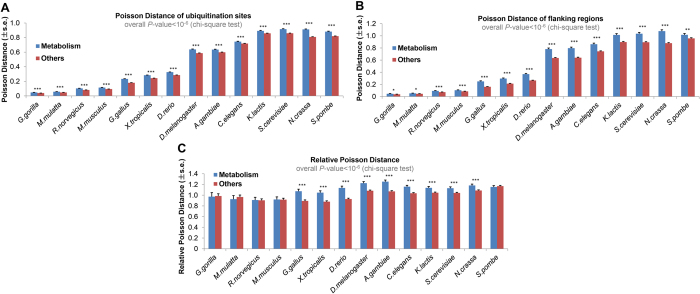
Constraints of KEGG pathway categories on ubiquitination sites conservation (s.e., standard error; *P-value < 0.05; **P-value < 0.01; ***P-value < 0.001). (**A**,**B**) Using Poisson distance to evaluate the conservation of ubiquitination sites/flanking regions in metabolism pathway and other pathways. (**C**) Using relative Poisson distance (see Materials and Methods) to compare the evolutionary conservations of ubiquitination sites between metabolism pathway and other pathways.

We mapped 8245 non-redundant human ubiquitination proteins with 35197 ubiquitination sites to 283 KEGG pathways^28^. These pathways were further grouped into six large functional categories: metabolism, genetic information processing, environmental information processing, cellular processes, organismal systems and human diseases. Using Poisson distance, we pair-wisely compared the evolutionary conservation of ubiquitination sites between six KEGG pathway categories. As a result, different KEGG pathway categories have different evolutionary conservations across multiple reference organisms (Fig. S1). In particular, ubiquitination sites involved in the metabolism pathway evolved at a higher rate than those in other pathways (Fig. 3A). However, we infer that this may be due to that proteins in metabolism pathway are more conserved during evolution regardless of ubiquitination. Therefore, we applied the same analysis to flanking regions, and found that flanking regions in metabolism pathway also evolve at a higher rate than those in other pathways (Fig. 3B). This finding confirms that protein conservation difference introduced by different protein categories cannot be ignored. To eliminate this influence, we calculated relative Poisson distance, which is the ratio of Poisson distance of the ubiquitination sites to that of their flanking regions in each group (see Materials and Methods). Using this relative Poisson distance, we found that ubiquitination sites in metabolism pathway show lower conservation compared with those in other pathways in multiple reference organisms (Fig. 3C). Furthermore, the small overall *P*-value across all the reference organisms demonstrates that the ubiquitination sites in the metabolism pathway (especially amino acid metabolism, metabolism of cofactors and vitamins, carbohydrate metabolism and lipid metabolism pathways. Fig. S2) evolved significantly more quickly than those in other pathways, which indicates that KEGG pathways like genetic information processing, environmental information processing, cellular processes, organismal systems and human diseases are likely to be constraints on the evolution of ubiquitination, while metabolism pathway is not much involved (it should be pointed out that ubiquitin mediated proteolysis is not included in KEGG metabolism pathway).

#### Gene Ontology terms

**Figure 4:**
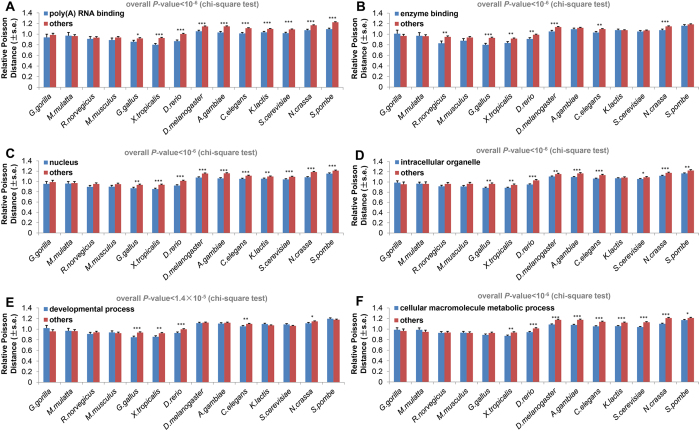
Constraints of certain Gene Ontology terms on ubiquitination sites conservation (s.e., standard error; *P-value < 0.05; **P-value < 0.01; ***P-value < 0.001). Relative Poisson distance of the ubiquitination sites for the Gene Ontology terms of poly (a) RNA binding (**A**), enzyme binding (**B**), nucleus (**C**), intracellular organelle (**D**), developmental process (**E**) and cellular macromolecule metabolic process (**F**).

We also classified ubiquitination proteins according to Gene Ontology terms^29^ and used relative Poisson distance to measure the evolutionary rate of ubiquitination sites. According to the result, ubiquitination sites in molecular function terms like poly (a) RNA binding (Fig. 4A), enzyme binding (Fig. 4B) and transcription factor binding (Fig. S3A) evolve with lower rates than those in other functional terms, while ubiquitination sites in term like oxidoreductase activity evolve with higher rate (Fig. S3B). These results suggest that ubiquitination plays more important roles in certain molecular functions. As for cellular component, we found that ubiquitination sites are more conserved in components of nucleus (Fig. 4C) and ribonucleoprotein complex (Fig. S3C) than in others, which suggests ubiquitination is important to these cellular components, this finding is in accordance with previous study^30^. In addition, we found that ubiquitination sites of intracellular organelle (Fig. 4D) have a lower evolutionary rate than those of other proteins, while ubiquitination sites of extracellular matrix have a higher evolutionary rate (Fig. S3D), which indicates ubiquitination is more important for intracellular proteins. For the ontology of biological process, result shows that ubiquitination sites in groups like developmental process (Fig. 4E) and cellular macromolecule metabolic process (Fig. 4F) are more conserved compared with those in other biological processes; on the contrary, ubiquitination sites in lipid metabolic process (Fig. S3D) and small molecule metabolic process (Fig. S3E) are less conserved than those in other biological processes. These findings are consistent with our previous hypothesis: ubiquitination sites are likely to participate in the fine regulation of cellular and developmental processes, and this improves the evolutionary conservation of ubiquitination sites during evolution.

#### Protein tissue expression specificity

An obvious correlate of pleiotropy in multicellular organisms is breadth of expression: proteins that are expressed in many tissues have to operate under diverse cellular conditions and might interact with diverse proteins. It has been proved that in mammals, insects and plants, broadly expressed proteins evolve more slowly than tissue-specific proteins^31^. Therefore, we anticipate that ubiquitination sites evolution might be affected by their protein tissue expression specificity.

**Figure 5:**
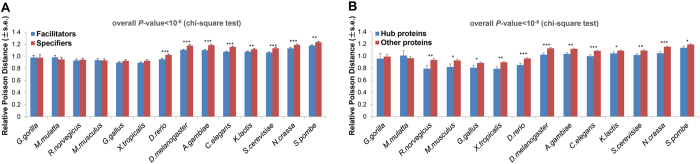
Constraint of protein tissue expression specificity and protein connectivity in protein-protein interaction network on ubiquitination sites conservation (s.e., standard error; *P-value < 0.05; **P-value < 0.01; ***P-value < 0.001). (**A**) Constraint of protein tissue expression specificity. The protein tissue expression specificity is measured by TSPS. Following Ravasi *et al*.^32^, we define proteins with widespread expression (TSPS < 1) as “facilitators”, and those with high tissue specificity (TSPS ≥ 1) as “specifiers”. (**B**) Constraint of protein connectivity in protein-protein interaction network. The protein connectivity is measured by protein degree (number of interaction partners in the human protein interaction network from HPRD Release 9). We define proteins with degree larger than 20 as hub proteins.

For each protein, we calculated its tissue-specificity score TSPS^32^ to measure the extent to which its expression distribution departs from the null distribution of uniform expression across all tissues. According to the definition of TSPS, a minimal TSPS = 0 means proteins uniform expression across all tissues, while a maximal TSPS ≈ 5 means only in a single tissue. Following Ravasi *et al*.^32^, we define proteins with widespread expression (TSPS < 1) as “facilitators”, and those with high tissue specificity (TSPS ≥ 1) as “specifiers”. Using relative Poisson distance, we found that ubiquitination sites in “facilitators” are more conserved compared with those in “specifiers” (Fig. 5A). This preference suggests that protein expression pattern can act as a constraint on ubiquitination site evolution and ubiquitination plays more important roles in broadly expressed “facilitators”.

#### Protein connectivity in protein-protein interaction network

Protein interaction networks are principal components of a system-level description of the cell^33^,^34^,^35^,^36^, and many previous studies have explored global aspects of network topology, clearly linking it to protein function, expression dynamics and other genomic features^37^,^38^,^39^,^40^. Here, we adopted protein degree (number of direct interaction partners) to measure protein connectivity in human protein-protein interaction network (HPRD^41^ Release 9), and we define proteins with degree larger than 20 as hub proteins. Using relative Poisson distance to evaluate ubiquitination sites conservation, we found that ubiquitination sites on hub proteins are more conserved than those on other proteins (Fig. 5B), which suggests that hub proteins exert stronger constraints on ubiquitination sites evolution. As hub proteins play essential roles in biological processes^42^, this constraint and subsequent adaptive evolution of ubiquitination sites increase the opportunity for ubiquitination to function widely.

In conclusion, by classifying ubiquitination sites into various categories instead of taking all ubiquitination sites as a whole, we found that various cellular and developmental processes, protein tissue expression specificity and protein connectivity in protein-protein interaction network can act as functional constraints on the evolution of ubiquitination sites. All these functional constraints increase the opportunity for the complex regulation of the ubiquitination-mediated cellular and developmental processes during evolution, leading to the promotion in evolutionary conservation of ubiquitination sites. Considering that functionally more important molecules, or portions of molecules, evolve slower than less important ones^43^, we think that ubiquitination play more important roles in these functional categories. These findings provide a novel view for the research of ubiquitination evolutionary events.

### Gains and functional distribution of novel ubiquitination sites during evolution

**Figure 6:**
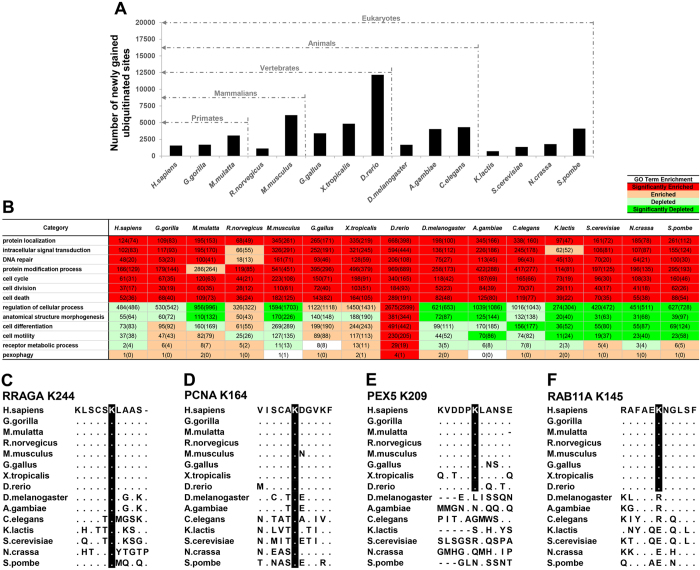
Gain of novel ubiquitination sites during evolution. (**A**) Detection and timing of gains of ubiquitination sites during evolution. The numbers of newly gained ubiquitination sites were plotted at different evolutionary time, and 14 reference organisms were arranged along the X axis in the order of their evolutionary distance to *H. sapiens*. Five key points of evolutionary divergence time (eukaryote, animal, vertebrate, mammalian and primate) with great promotion of ubiquitination sites were marked by the dashed line. (**B**) Functional distribution of newly gained ubiquitination lysines. GOfact^54^ was used to calculate the category enrichment for the newly gained ubiquitination proteins. Significant enrichment/depletion was defined as *P*-value < 0.05 (hyper geometry distribution test). The number in each table cell is the number of observed proteins in certain functional category, and the number in brackets is the random expectation. (**C**–**F**) Four examples of gains of ubiquitination sites. Central ubiquitination lysines and the surrounding regions for proteins RRAGA, PCNA, PEX5 and RAB11A are shown based on multiple sequence alignment. The gained ubiquitination sites are painted in black. The residues that are the same as those in human sequence are represented as dots (.). Dash (−) is alignment gap.

The above analysis suggests that certain biological functions impose constraints on ubiquitination sites evolutionary conservation, and we wonder whether the emergence of ubiquitination sites at different evolutionary time were affected by these functional constraints. To answer this question, we performed multiple sequence alignment to identify ubiquitination sites emerged at different evolutionary time (Fig. 6A), and functional enrichment analysis to explore the functional characters of these corresponding ubiquitination proteins (Fig. 6B). We have listed all the cases of newly gained ubiquitination sites at different evolutionary stages in Supplementary Table S1.

As shown in Fig. 6A, at the divergence time of eukaryote, ubiquitination sites begin to emerge with large amount, indicating that ubiquitination starts to play important roles in eukaryotic biological processes. There follows four notable incensements of ubiquitination sites at the divergence time of animal, vertebrate, mammalian and primate respectively, which are all critical evolutionary points. This phenomenon is supposed to promote the complex regulation of ubiquitination-mediated cellular and developmental processes.

From Fig. 6B, we found that at the divergence time of eukaryote, there gains a significantly improved portion of ubiquitination sites for the process of protein modification. This finding is consistent with the phenomenon that ubiquitination mediates 80~85% of the protein degradation in eukaryotic cells (although ubiquitin like protein Pup exist in prokaryote, it doesn’t play as important role of protein degradation as in eukaryotic cells^44^). We also found that proteins of newly gained ubiquitination sites at divergence time of eukaryote are enriched in function categories like cell cycle, cell division, cell death, protein localization, intracellular signal transduction and DNA repair. For example, K244 ubiquitination site of protein RRAGA emerged at divergence time of eukaryote (Fig. 6C). The K244 was found to be attached by a K63-linked ubiquitin chain mediated by RNF152 and this event can regulate mTORC1 signal transduction^45^. Another example (Fig. 6D) is mono-ubiquitination of PCNA on K164 (gained at eukaryote), which is essential for DNA repair by translation DNA synthesis^46^.

At the divergence time of vertebrate, there are great proportions of newly gained ubiquitination sites enriched in functions like regulation of cellular process, cell differentiation, anatomical structure morphogenesis, cell motility, receptor metabolic process and pexophagy, which are typical cellular and developmental processes. For example, PEX5 gained a ubiquitination site K209 at vertebrate (Fig. 6E), this ubiquitination site is recognized by the autophagy adaptor protein p62, directing the autophagosome to peroxisomes to induce pexophagy^47^. Another example is that co-expression of ADRB2 (Beta-2 adrenergic receptor) with HACE1 induces the ubiquitination and activation of Rab11a at K145 (gained at vertebrate) (Fig. 6F), which in turn regulates ADRB2 receptor metabolic process^48^.

We found that during evolution, the proteins containing newly gained ubiquitination sites are significantly enriched in the functions of protein localization, protein modification, cell cycle, cell differentiation and anatomical structure morphogenesis, which are all fundamental cellular and developmental processes for the survival of the cell. This finding validates our hypothesis that the emergences of ubiquitination sites at different evolutionary time were also affected by multiple functional constraints. This suggests that ubiquitination events play more and more important roles in the fine-tuning mechanism of cell survival in the whole process of evolution.

As Hagai *et al*. pointed out, there exist three levels of ubiquitination sites evolutionary conservation: conserved, compensated and uncompensated, while the latter two belong to the situation of non-conserved ubiquitination sites^14^. In this paper, we only focus on the strict definition of conservation, trying to answer the question that whether the ubiquitination sites are conserved along the evolution and what the functional constrains are. Therefore, we didn’t perform ubiquitination sites compensation analysis in this study, although the compensation mechanism is also very important for ubiquitination sites evolution^14^. In addition, it is impossible for us to implement the statistical evolutionary analysis for this compensation mechanism now, because there is not enough functional annotation information available to determine whether a nearby lysine is functionally compensated or not.

## Materials and Methods

### Preprocessing Data

Datasets of human ubiquitination proteins and sites were retrieved from PhosphoSitePlus^49^. Protein sequences were downloaded from UniProtKB^50^. The orthologs of the ubiquitination proteins in *G. gorilla, M. mulatta, R. norvegicus, M. musculus, G. gallus, X. tropicalis, D. rerio, D. melanogaster, A. gambiae, C. elegans, K. lactis, S. cerevisiae, N. crassa* and *S. pombe* were downloaded from Inparanoid V8.0^51^. Clustal Omega^52^ was used for sequence alignment, and the orthologous sites of the human ubiquitination sites were extracted from the alignments. Local protein structures were predicted by the VSL2 software^53^. Pathway assignment was performed based on the KEGG database^28^ (Release 53.0). The enrichment of specific GO terms was tested using a hyper geometry distribution test, followed by the Bonferroni multiple testing correction to control for the false discovery rate^54^. Protein-protein interaction data were retrieved from HPRD^41^ (Release 9). Protein tissue expression data were downloaded from ProteomicsDB^55^ (20150420).

### Calculating Poisson distance

For each ubiquitination protein in human, we retrieved its orthologous protein from the reference organisms. Then we used Clustal Omega to align the sequence of human ubiquitination protein and that of its ortholog. The orthologous site of human protein ubiquitination site was extracted from the alignments to estimate whether the ubiquitination site is remained or changed in reference organism.

For a protein group *i* in human, we calculated the proportion of different residues between the ubiquitination sites of human protein and their orthologous sites in organism *o* as *p*_*i,o*_, which is used for comparing the extent of homologous ubiquitination sites divergence:


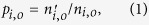


where 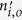 denotes the number of different residues at the ubiquitination site and *n*_*i,o*_ denotes the number of all residues. Here, 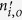 follows binomial distribution, therefore, the variance of *p*_*i,o*_ is given by


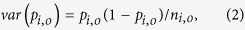


as the reference Nei and Kumar^16^, we took the Poisson correction to transform variance proportion *p*_*i,o*_ value into Poisson distance *d*_*i,o*_ to measure the degree of conservation, which can correct multiple substitutions at a site and has the linear relationship with time.


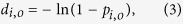


then the variance of 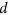 is given by


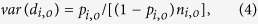


and then, Poisson distance *d*_*i,o*_ will be applied to measure the evolutionary distance of ubiquitination sites between human and reference organism. The larger the Poisson distance, the higher the evolutionary rate and the lower the evolutionary conservation.

### Contrasting ubiquitination sites with their flanking regions

Referring to phosphorylation sites evolution analysis^17^,^18^, we selected the ten flanking residues around a ubiquitination site rather than other lysine sites or random sample of sites as the background based on following speculations. PTM (post-translational modification) sites can be conserved for many other reasons rather than ubiquitination alone, such as protein dispensability, expression abundance, and others^31^. Besides, ubiquitination sites locate preferentially in disordered regions, the local structure will impose constraints on the conservation of ubiquitination sites^56^,^57^. Therefore comparing ubiquitination sites with a random sample of sites or other lysine residues can be misleading. Whether ubiquitination sites are more conserverd than non-ubiquitination sites can only be estimated when local protein structures are taken into account^17^,^18^,^58^.

The flanking regions were defined as the ten residues centered on a ubiquitination site. All flanking regions in a protein category *i* were extracted and combined together, and the Poisson distance of the flanking regions was estimated with the same method as we did for the ubiquitination sites. The z-score was used to assess whether the ubiquitination sites and the flanking regions evolved at the same rate^17^, which is calculated as:


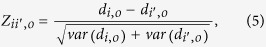


where *d*_*i,o*_ and *d*_*i*′,*o*_ are the Poisson distance for the ubiquitination sites and the flanking regions, respectively. Under the null hypothesis, the z-score follows the standard normal distribution approximately. The obtained z-score *Z*_*ii*′,*o*_ can be used to contrast ubiquitination sites with flanking regions in a single organism *o*. To compare the evolutionary conservation of the ubiquitination sites with that of flanking regions in the protein category *i* across multiple organisms, we further calculate:


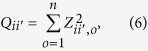


which follows a chi-square distribution with the degree of freedom equal to *n (n* denotes the number of organisms considered).

### Calculating relative Poisson distance

We introduced relative Poisson distance to eliminate difference of evolutionary conservations between protein categories caused by protein intrinsic properties. For reference organism *o*, the relative Poisson distance of ubiquitination sites in protein category *i* is calculated as:


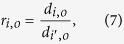


where *d*_*i,o*_ and *d*_*i*′,*o*_ denote the Poisson distance of ubiquitination sites and flanking regions in protein category *i*, respectively. The estimate variance will be:


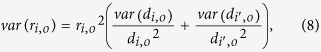


where *var(d*_*i,o*_) and *var(d*_*i*′,*o*_) represent estimated variance for the Poisson distance of ubiquitination sites and flanking regions in protein category *i*, respectively. The larger the relative Poisson distance, the higher the evolutionary rate and the lower the evolutionary conservation.

### Comparing ubiquitination sites between categories

The z-score was also calculated to assess the difference of relative Poisson distance between two categories *i* and *j*:


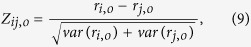


where *r*_*i,o*_ and *r*_*j,o*_ denote the relative Poisson distance of ubiquitination sites in protein categories *i* and *j* respectively, *var(r*_*i,o*_) and *var(r*_*j,o*_) represent estimated variance for the relative Poisson distance of ubiquitination sites in protein categories *i* and *j* respectively. The z-score follows the standard normal distribution approximately. The obtained z-score *Z*_*ij,o*_ reflects the difference of ubiquitination sites evolutionary conservation between the categories *i* and *j* in a single organism *o*. To assess the difference of ubiquitination sites conservation between the categories *i* and *j*across multiple organisms, we further calculate:


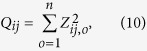


which follows a chi-square distribution with the degree of freedom equal to *n(n* denotes the number of organisms considered) under the null hypothesis.

### Calculating protein tissue expression specificity score

Using relative entropy, protein tissue expression specificity score *TSPS*_*i*_^32^ is calculated as:


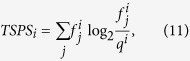


where 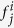 (the fractional expression level of the protein *i* expression level in tissue *j*) is computed as the ratio of the protein *i*expression level in tissue *j* to its sum total expression level across all tissues, and *q*^*i*^ was the fractional expression of protein *i* under a null model assuming uniform expression across tissues.

## Additional Information

**How to cite this article**: Lu, L. *et al*. Functional constraints on adaptive evolution of protein ubiquitination sites. *Sci. Rep.* **7**, 39949; doi: 10.1038/srep39949 (2017).

**Publisher's note:** Springer Nature remains neutral with regard to jurisdictional claims in published maps and institutional affiliations.

## Supplementary Material

Supplementary Information

Supplementary Dataset 1
