# Supplementary material for: Functional constraints on adaptive evolution of protein ubiquitination sites
Source: Sci Rep. 2017 Jan 5;7:39949. doi: 10.1038/srep39949 (PMC5215434; doi:10.1038/srep39949)
Supplement: Supplementary Information [file srep39949-s1.pdf]

Supplementary Figures:
Functional constraints on adaptive evolution of protein ubiquitination sites

Liang Lu[1,2,3,4+], Yang Li[1,2,+], Zhongyang Liu[1,2], Fengji Liang[3,4], Feifei Guo[1,2], Shuai Yang[1,2], Dan Wang[1,2], Yangzhige He[1,2], Jianghui Xiong[3,4*], Dong Li[1,2,*] and Fuchu He[1,2,*]

[1]State Key Laboratory of Proteomics, Beijing Proteome Research Center, Beijing Institute of Radiation Medicine, 27 Taiping Road, Beijing 100850, China

[2]National Center for Protein Sciences Beijing, 38 Life Science Park Road, Beijing 102206, China

[3]State Key Laboratory of Space Medicine Fundamentals and Application, China Astronaut Research and Training Center, 26 Beiqing Road, Beijing 100094, China

[4]Space Institute of Southern China, 3 Pingdi Industrial Road, Shenzhen 518117, China

[+]These authors contributed equally to this work.

[*]**Corresponding authors:** xiongjh77@163.com; lidong.bprc@foxmail.com; hefc@nic.bmi.ac.cn

Supplementary Figure S1

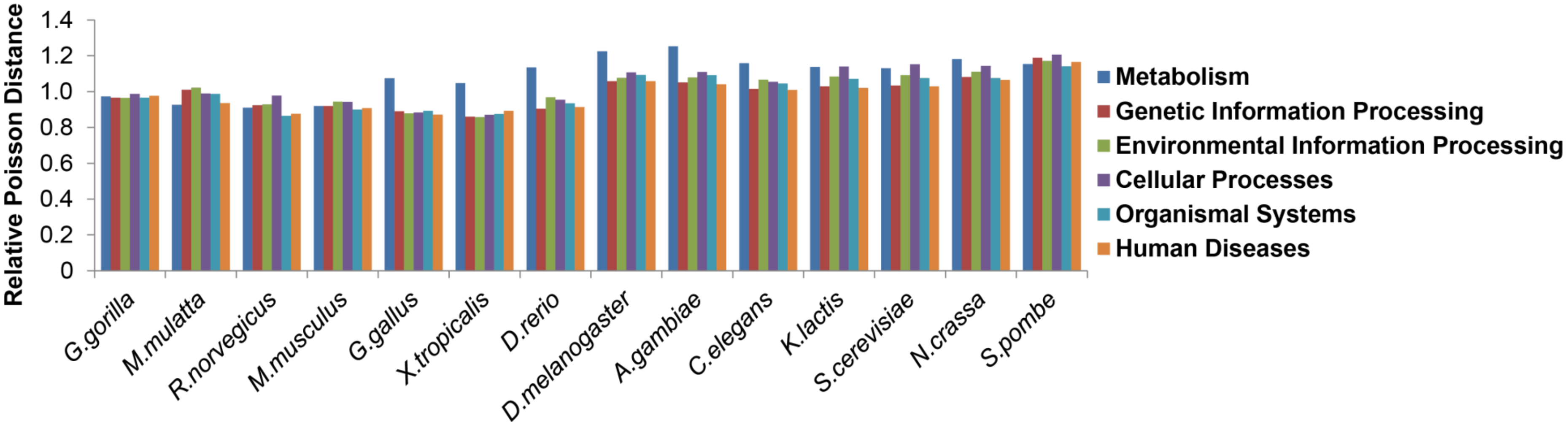

Supplementary Figure S1. Constraints of KEGG pathway categories on ubiquitination sites conservation. Poisson distance was used to evaluate conservation of ubiquitination sites in six large KEGG pathways of metabolism, genetic information processing, environmental information processing, cellular processes, organismal systems and human diseases.

Supplementary Figure S2

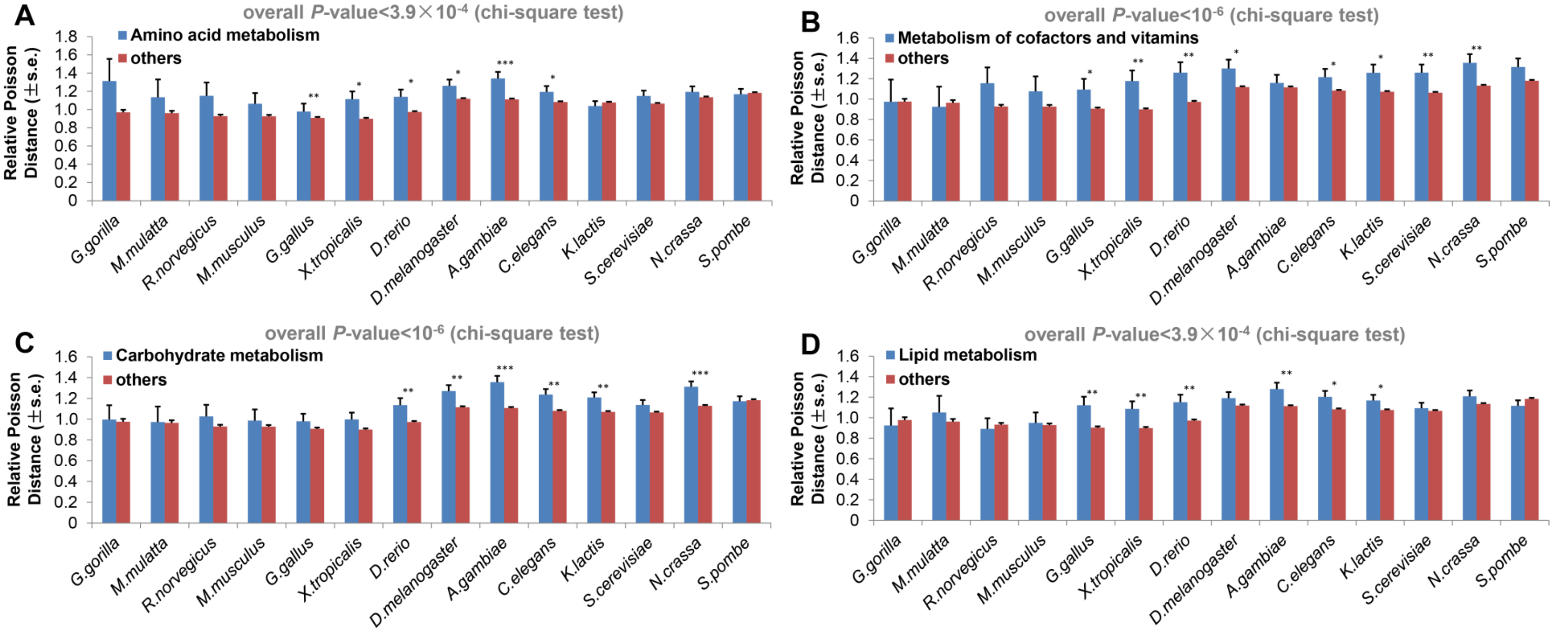

Supplementary Figure S2. Constraints of sub categories of KEGG metabolism pathway on ubiquitination sites conservation (s.e., standard error; *, *P*-value<0.05; **, *P*-value<0.01; *, *P*-value<0.001).** Relative Poisson distance of the ubiquitination sites for the KEGG metabolism pathways of amino acid metabolism (A), metabolism of cofactors and vitamins (B), carbohydrate metabolism (C) and lipid metabolism (D).

Supplementary Figure S3

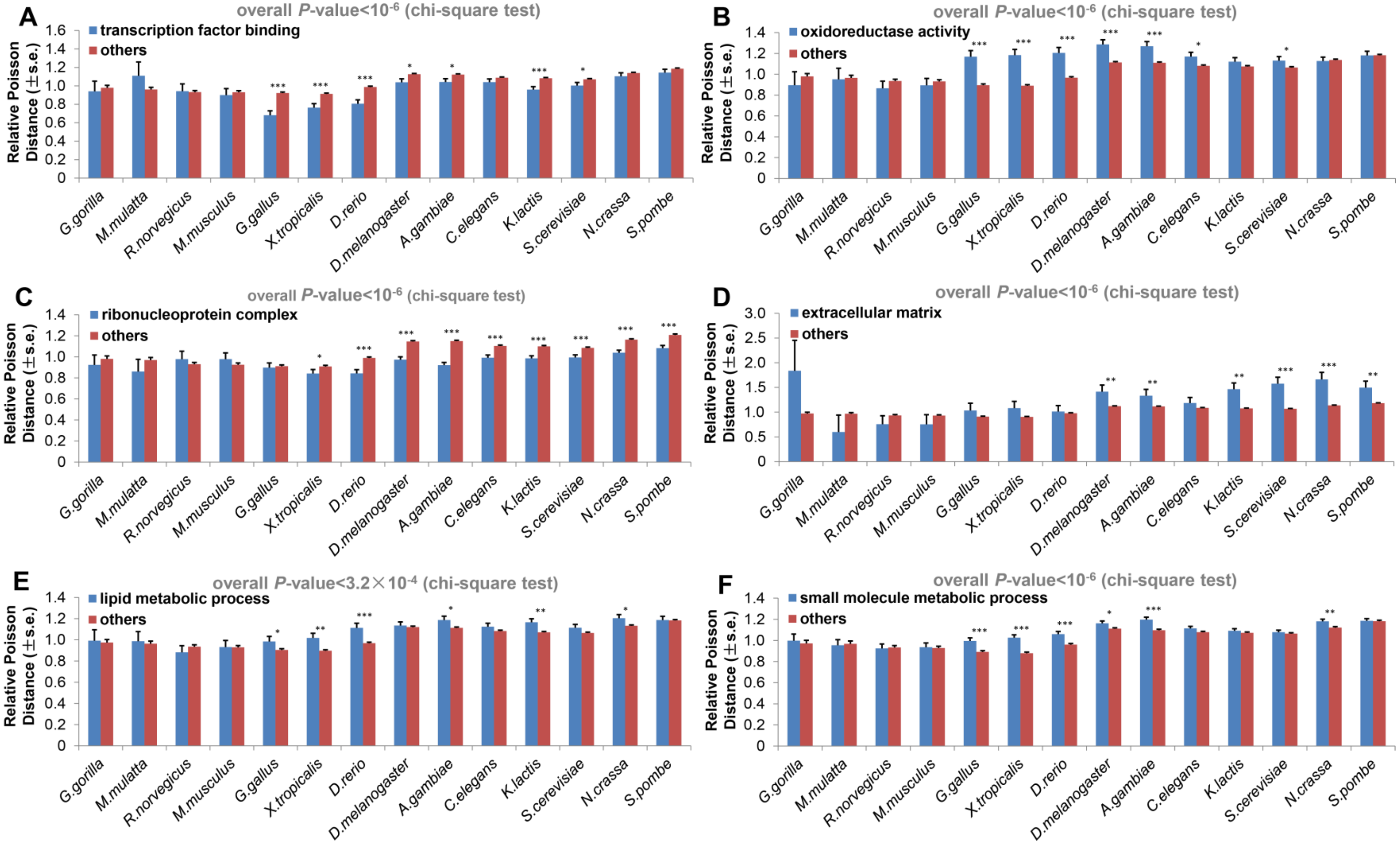

Figure S3. Constraints of certain Gene Ontology terms on ubiquitination sites conservation (s.e., standard error; *, *P*-value<0.05; **, *P*-value<0.01; *, *P*-value<0.001).** Relative Poisson distance of the ubiquitination sites for the Gene Ontology terms of transcription factor binding (A), oxidoreductase activity (B), ribonucleoprotein complex (C), extracellular matrix (D), lipid metabolic process (E) and small molecule metabolic process (F).
